# Supplementary material for: A Likelihood Approach for Real-Time Calibration of Stochastic Compartmental Epidemic Models
Source: PLoS Comput Biol. 2017 Jan 17;13(1):e1005257. doi: 10.1371/journal.pcbi.1005257 (PMC5240920; doi:10.1371/journal.pcbi.1005257)
Supplement: S1 File — (TAR.GZ) [file pcbi.1005257.s014.tar.gz › HSPH_Online-SI-Revision/PF08/n100-extreme/n100-extreme_PF2_table-4.pdf]

1	newly 3 weeks spec	10683.	5963.86	{5829., 6083.}	44.1743	0.253239	newly 3 weeks cumul	25079.	13927.	{13704., 14095.}	44.4676	0.255484	newly 5 weeks spec	10855.	7789.77	{7634., 7948.}	28.238	0.14416	newly 5 weeks cumul	47373.	28816.6	{28438., 29224.}	39.1708	0.215915	newly 58 weeks cumul	85292.	79016.2	{78650., 79358.}	7.35808	0.0331948
2	newly 3 weeks spec	8383.	6500.53	{6165., 6716.}	22.4558	0.110693	newly 3 weeks cumul	21608.	16893.1	{15860., 17379.}	21.8204	0.107151	newly 5 weeks spec	8027.	7025.63	{6770., 7194.}	12.475	0.0580333	newly 5 weeks cumul	38163.	30896.9	{29279., 31650.}	19.0396	0.0919199	newly 54 weeks cumul	72415.	75797.	{75414., 76674.}	4.8654	0.0206044
3	newly 3 weeks spec	12915.	9237.81	{8897., 9530.}	28.4722	0.145697	newly 3 weeks cumul	29820.	21361.4	{20574., 22002.}	28.3656	0.145046	newly 5 weeks spec	11421.	10236.6	{10031., 10433.}	10.3706	0.0476078	newly 5 weeks cumul	54424.	41912.8	{40664., 43033.}	22.9884	0.113538	newly 59 weeks cumul	84212.	88611.	{87737., 89201.}	5.22367	0.0221044
4	newly 3 weeks spec	15590.	6782.11	{4778., 9849.}	56.497	0.384127	newly 3 weeks cumul	27674.	12942.5	{9338., 18580.}	53.2323	0.349774	newly 5 weeks spec	15345.	10937.6	{8797., 13888.}	28.7223	0.155559	newly 5 weeks cumul	61225.	33353.3	{25241., 45330.}	45.5235	0.278551	newly 63 weeks cumul	88766.	89222.4	{81436., 92641.}	5.24198	0.0229618
5	newly 3 weeks spec	10521.	6725.81	{6308., 7101.}	36.0725	0.194776	newly 3 weeks cumul	21016.	14191.7	{13304., 15090.}	32.4721	0.170964	newly 5 weeks spec	12782.	9780.23	{9406., 10070.}	23.4844	0.116407	newly 5 weeks cumul	46785.	32608.1	{30908., 34097.}	30.3023	0.157054	newly 60 weeks cumul	85329.	90596.3	{89867., 91235.}	6.17287	0.0260068
6	newly 3 weeks spec	10702.	5381.38	{5226., 5531.}	49.7161	0.298701	newly 3 weeks cumul	23963.	12285.4	{11985., 12576.}	48.7318	0.290238	newly 5 weeks spec	11271.	7529.24	{7334., 7718.}	33.1981	0.175309	newly 5 weeks cumul	47247.	26480.1	{25898., 26966.}	43.9539	0.251528	newly 58 weeks cumul	86171.	80385.8	{80074., 80718.}	6.71362	0.0301847
7	newly 3 weeks spec	11555.	4082.31	{3975., 4194.}	64.6706	0.45196	newly 3 weeks cumul	26529.	9636.23	{9466., 9806.}	63.6766	0.439856	newly 5 weeks spec	11237.	5756.65	{5622., 5901.}	48.7706	0.290573	newly 5 weeks cumul	49918.	20380.7	{19966., 20738.}	59.1716	0.389085	newly 56 weeks cumul	84780.	80161.5	{79740., 80572.}	5.44759	0.0243314
8	newly 3 weeks spec	11018.	5351.79	{5173., 5510.}	51.4268	0.31374	newly 3 weeks cumul	24447.	12351.3	{12055., 12600.}	49.4772	0.296598	newly 5 weeks spec	11207.	7353.18	{7174., 7515.}	34.3876	0.183093	newly 5 weeks cumul	47781.	26235.8	{25667., 26733.}	45.0915	0.260425	newly 58 weeks cumul	87124.	79130.4	{78669., 79569.}	9.17501	0.0417988
9	newly 3 weeks spec	10870.	4408.93	{4194., 4584.}	59.4395	0.392339	newly 3 weeks cumul	22125.	9970.11	{9466., 10321.}	54.9374	0.346554	newly 5 weeks spec	12694.	6596.81	{6369., 6821.}	48.0321	0.284502	newly 5 weeks cumul	47679.	22155.2	{21262., 22861.}	53.5326	0.333141	newly 58 weeks cumul	88989.	80708.6	{80237., 81117.}	9.30493	0.0424233
10	newly 3 weeks spec	15442.	7092.54	{6729., 7500.}	54.0698	0.33824	newly 3 weeks cumul	29673.	14592.	{13927., 15467.}	50.8239	0.30862	newly 5 weeks spec	11765.	10426.	{10168., 10702.}	11.3816	0.0526509	newly 5 weeks cumul	57244.	34262.7	{33251., 35616.}	40.1462	0.223133	newly 62 weeks cumul	72720.	91078.1	{91031., 92424.}	25.2449	0.0975779
11	newly 3 weeks spec	9197.	7282.72	{7132., 7423.}	20.8142	0.101427	newly 3 weeks cumul	23145.	18248.8	{17906., 18577.}	21.1545	0.103285	newly 5 weeks spec	8893.	8003.83	{7838., 8149.}	9.99854	0.0457955	newly 5 weeks cumul	41559.	34176.2	{33693., 34647.}	17.7646	0.0849794	newly 56 weeks cumul	75767.	78020.5	{77679., 78306.}	2.97421	0.012724
12	newly 3 weeks spec	11654.	3913.44	{3800., 4034.}	66.4198	0.47408	newly 3 weeks cumul	24296.	8992.22	{8804., 9189.}	62.9889	0.431758	newly 5 weeks spec	12745.	5847.	{5642., 6014.}	54.1232	0.338534	newly 5 weeks cumul	50388.	19766.2	{19271., 20218.}	60.772	0.406489	newly 57 weeks cumul	88506.	81778.9	{78970., 82578.}	7.60078	0.0343836
13	newly 3 weeks spec	8371.	4393.49	{4279., 4521.}	47.5154	0.280079	newly 3 weeks cumul	19985.	10781.3	{10600., 10967.}	46.053	0.268077	newly 5 weeks spec	8948.	5639.67	{5489., 5749.}	36.9728	0.200552	newly 5 weeks cumul	38014.	21527.2	{21136., 21884.}	43.3704	0.246999	newly 55 weeks cumul	83218.	73323.4	{72855., 73789.}	11.89	0.0549795
14	newly 3 weeks spec	11434.	1505.03	{1099., 1933.}	86.8372	0.892414	newly 3 weeks cumul	18644.	3301.09	{2552., 4092.}	82.2941	0.76122	newly 5 weeks spec	15310.	2769.45	{1897., 3612.}	81.9108	0.757494	newly 5 weeks cumul	50732.	8132.98	{5842., 10426.}	83.9687	0.807013	newly 60 weeks cumul	70240.	79153.4	{74084., 83594.}	13.6282	0.0552113
15	newly 3 weeks spec	13412.	7395.89	{6946., 7735.}	44.8562	0.258932	newly 3 weeks cumul	27516.	15861.6	{14840., 16654.}	42.3549	0.23968	newly 5 weeks spec	12056.	10086.1	{9762., 10335.}	16.3397	0.0776213	newly 5 weeks cumul	54113.	35162.9	{33391., 36386.}	35.0196	0.187482	newly 60 weeks cumul	75951.	90175.2	{89503., 90789.}	18.7281	0.0745454
16	newly 3 weeks spec	15726.	6061.23	{5910., 6238.}	61.4573	0.414146	newly 3 weeks cumul	30526.	12574.8	{12291., 12850.}	58.8063	0.385233	newly 5 weeks spec	14011.	9482.	{9242., 9660.}	32.3246	0.169636	newly 5 weeks cumul	61514.	30130.1	{29427., 30662.}	51.0191	0.310028	newly 60 weeks cumul	88752.	90984.3	{90652., 91258.}	2.51523	0.0107869
17	newly 3 weeks spec	9053.	6263.13	{6120., 6397.}	30.8171	0.160062	newly 3 weeks cumul	21971.	15630.4	{15313., 15896.}	28.8591	0.147928	newly 5 weeks spec	9419.	7265.07	{7125., 7394.}	22.8679	0.112803	newly 5 weeks cumul	40887.	29855.6	{29486., 30232.}	26.9802	0.136586	newly 55 weeks cumul	79924.	79478.5	{79156., 79849.}	0.574784	0.00250588
18	newly 3 weeks spec	8959.	3093.55	{2979., 3203.}	65.4699	0.46196	newly 3 weeks cumul	19558.	7421.62	{7305., 7568.}	62.0533	0.420871	newly 5 weeks spec	10392.	4372.51	{4265., 4469.}	57.9243	0.376049	newly 5 weeks cumul	40376.	15542.8	{15263., 15808.}	61.5048	0.414638	newly 56 weeks cumul	75084.	72410.2	{72029., 72746.}	3.56102	0.0157513
19	newly 3 weeks spec	11412.	4703.36	{4566., 4834.}	58.7858	0.385057	newly 3 weeks cumul	23492.	10569.9	{10408., 10726.}	55.0064	0.346897	newly 5 weeks spec	12862.	6998.55	{6844., 7162.}	45.5874	0.264392	newly 5 weeks cumul	49674.	23522.4	{23192., 23907.}	52.6465	0.324692	newly 58 weeks cumul	89565.	81841.1	{81527., 82166.}	8.62378	0.0391707
20	newly 3 weeks spec	15824.	756.07	{290., 1581.}	95.222	1.40671	newly 3 weeks cumul	21990.	1572.7	{698., 3015.}	92.8481	1.21194	newly 5 weeks spec	20039.	1702.53	{486., 4106.}	91.5039	1.20561	newly 5 weeks cumul	66304.	4429.77	{1562., 9665.}	93.319	1.27621	newly 63 weeks cumul	91232.	76885.4	{63525., 91905.}	16.0403	0.0796281
21	newly 3 weeks spec	11565.	7327.01	{6924., 7756.}	36.645	0.198583	newly 3 weeks cumul	23774.	15628.3	{14829., 16580.}	34.2629	0.182541	newly 5 weeks spec	12361.	10124.5	{9802., 10379.}	18.0933	0.0868047	newly 5 weeks cumul	49979.	34945.5	{33379., 36244.}	30.0796	0.155609	newly 60 weeks cumul	80337.	90490.1	{89899., 91025.}	12.6381	0.0516777
22	newly 3 weeks spec	13043.	7766.33	{7517., 7994.}	40.456	0.225289	newly 3 weeks cumul	25673.	16340.7	{15785., 16818.}	36.3506	0.19634	newly 5 weeks spec	13126.	10638.1	{10487., 10815.}	18.9544	0.0912999	newly 5 weeks cumul	53143.	36743.3	{35931., 37369.}	30.8596	0.160318	newly 61 weeks cumul	81637.	91328.3	{91081., 91539.}	11.8712	0.0487177
23	newly 3 weeks spec	8971.	3845.24	{3728., 3943.}	57.137	0.368027	newly 3 weeks cumul	22691.	9666.63	{9493., 9815.}	57.3988	0.370615	newly 5 weeks spec	8717.	4920.48	{4786., 5052.}	43.5531	0.248455	newly 5 weeks cumul	40575.	19035.6	{18739., 19356.}	53.0854	0.328732	newly 54 weeks cumul	74886.	72614.2	{72272., 73007.}	3.03369	0.013383
24	newly 3 weeks spec	9753.	3601.88	{3495., 3697.}	63.069	0.432727	newly 3 weeks cumul	22644.	8962.59	{8804., 9127.}	60.4196	0.40257	newly 5 weeks spec	10377.	4703.31	{4566., 4830.}	54.6756	0.343765	newly 5 weeks cumul	43672.	17861.3	{17479., 18182.}	59.1014	0.388346	newly 54 weeks cumul	86096.	72700.6	{72300., 73141.}	15.5586	0.073449
25	newly 3 weeks spec	14475.	2840.31	{2722., 2922.}	80.3778	0.707406	newly 3 weeks cumul	28620.	6583.78	{6482., 6680.}	76.9959	0.638231	newly 5 weeks spec	13481.	4402.61	{4274., 4538.}	67.3421	0.486124	newly 5 weeks cumul	57956.	14597.8	{14351., 14858.}	74.8124	0.598859	newly 57 weeks cumul	86463.	79217.7	{78885., 79603.}	8.37971	0.0380108
26	newly 3 weeks spec	8715.	6789.94	{6652., 6940.}	22.089	0.108517	newly 3 weeks cumul	21621.	17079.6	{16747., 17409.}	21.0048	0.102515	newly 5 weeks spec	8698.	7619.56	{7475., 7758.}	12.3987	0.0575506	newly 5 weeks cumul	39423.	32151.5	{31746., 32648.}	18.4449	0.0886186	newly 55 weeks cumul	81661.	80024.	{79600., 80408.}	2.00462	0.00879759
27	newly 3 weeks spec	9364.	7191.05	{7040., 7385.}	23.2054	0.114756	newly 3 weeks cumul	24919.	18346.2	{17969., 18677.}	26.3767	0.133038	newly 5 weeks spec	8776.	7663.28	{7512., 7810.}	12.6791	0.058934	newly 5 weeks cumul	43318.	33708.3	{33188., 34207.}	22.1842	0.108966	newly 56 weeks cumul	79621.	74698.5	{74242., 75717.}	6.18241	0.0277255
28	newly 3 weeks spec	11755.	9968.92	{9676., 10211.}	15.1942	0.0716704	newly 3 weeks cumul	27332.	23320.7	{22059., 23991.}	14.6764	0.0691346	newly 5 weeks spec	11093.	10470.	{10282., 10642.}	5.61661	0.0251498	newly 5 weeks cumul	50746.	44632.5	{43625., 45393.}	12.0473	0.0558047	newly 60 weeks cumul	87654.	89132.4	{88674., 89864.}	1.68668	0.00725942
29	newly 3 weeks spec	8104.	3867.01	{3773., 3952.}	52.2827	0.321393	newly 3 weeks cumul	20099.	9636.35	{9498., 9787.}	52.0556	0.319291	newly 5 weeks spec	8778.	5013.57	{4882., 5113.}	42.8848	0.243313	newly 5 weeks cumul	37699.	19147.1	{18861., 19431.}	49.2105	0.294254	newly 54 weeks cumul	75236.	73701.5	{73356., 74009.}	2.03962	0.00895286
30	newly 3 weeks spec	13462.	9352.3	{8741., 9945.}	30.5282	0.159252	newly 3 weeks cumul	25138.	17819.6	{16736., 18973.}	29.1128	0.150466	newly 5 weeks spec	14527.	13234.3	{12981., 13610.}	8.89833	0.040711	newly 5 weeks cumul	55394.	43452.3	{41454., 45418.}	21.5577	0.106044	newly 63 weeks cumul	91285.	93156.1	{92928., 93365.}	2.04972	0.00881107
31	newly 3 weeks spec	13467.	4624.31	{2079., 7676.}	65.6619	0.521469	newly 3 weeks cumul	21982.	8338.59	{4046., 13355.}	62.0663	0.467363	newly 5 weeks spec	17282.	9438.06	{4674., 14143.}	45.3879	0.305043	newly 5 weeks cumul	58272.	25060.7	{12120., 39467.}	56.9936	0.414067	newly 64 weeks cumul	87865.	87995.4	{81533., 93595.}	6.82417	0.0302975
32	newly 3 weeks spec	11691.	8116.26	{7914., 8304.}	30.5769	0.158581	newly 3 weeks cumul	23586.	17187.5	{16732., 17600.}	27.1284	0.137543	newly 5 weeks spec	12032.	10789.9	{10616., 10943.}	10.3232	0.04735	newly 5 weeks cumul	48919.	38000.	{37414., 38579.}	22.3206	0.109728	newly 61 weeks cumul	74173.	91687.7	{91410., 91962.}	23.6133	0.0920639
33	newly 3 weeks spec	9366.	8259.53	{7823., 8672.}	11.8137	0.0550139	newly 3 weeks cumul	20595.	18463.4	{17528., 19563.}	10.3503	0.0478689	newly 5 weeks spec	10722.	10142.1	{9943., 10362.}	5.4086	0.0242847	newly 5 weeks cumul	42102.	38328.9	{37019., 40000.}	8.9619	0.041027	newly 59 weeks cumul	87634.	89281.8	{89133., 89810.}	2.27736	0.00989396
34	newly 3 weeks spec	6522.	5355.33	{5213., 5518.}	17.8882	0.0857142	newly 3 weeks cumul	16514.	13549.	{13227., 13896.}	17.9546	0.0860295	newly 5 weeks spec	7135.	6260.67	{6083., 6430.}	12.2541	0.0568605	newly 5 weeks cumul	30872.	25771.	{25236., 26381.}	16.5232	0.0785027	newly 54 weeks cumul	67088.	72703.1	{71976., 73314.}	8.36981	0.0348986
35	newly 3 weeks spec	10120.	4599.93	{4135., 4830.}	54.5461	0.34333	newly 3 weeks cumul	20852.	10149.1	{9067., 10543.}	51.3278	0.31354	newly 5 weeks spec	12158.	7125.46	{6556., 7422.}	41.3928	0.232856	newly 5 weeks cumul	45344.	23234.6	{21162., 24120.}	48.7593	0.291191	newly 59 weeks cumul	90553.	80586.4	{79613., 81250.}	11.0064	0.0507579
36	newly 3 weeks spec	8518.	7279.02	{6812., 7554.}	14.5454	0.0685449	newly 3 weeks cumul	20967.	18024.7	{17138., 18691.}	14.0332	0.0658999	newly 5 weeks spec	8858.	8107.16	{7763., 8343.}	8.47641	0.0386214	newly 5 weeks cumul	39044.	34134.	{32445., 35192.}	12.5756	0.0585539	newly 57 weeks cumul	75101.	76538.4	{75945., 77058.}	1.91401	0.00822698
37	newly 3 weeks spec	14436.	7047.05	{6798., 7314.}	51.1842	0.311693	newly 3 weeks cumul	27190.	14450.7	{14071., 15220.}	46.8531	0.274823	newly 5 weeks spec	14344.	10447.2	{10245., 10645.}	27.1668	0.137722	newly 5 weeks cumul	57920.	34114.	{33389., 34994.}	41.1015	0.230007	newly 62 weeks cumul	85971.	91599.3	{90869., 92320.}	6.54674	0.027531
38	newly 3 weeks spec	14671.	2405.33	{1260., 3043.}	83.6049	0.80725	newly 3 weeks cumul	23153.	4743.73	{2616., 5911.}	79.5114	0.706071	newly 5 weeks spec	16782.	5285.39	{2611., 6602.}	68.5056	0.529235	newly 5 weeks cumul	59731.	13736.4	{7076., 17053.}	77.0029	0.661339	newly 62 weeks cumul	80717.	88200.2	{81933., 91995.}	12.0404	0.0504359
39	newly 3 weeks spec	8107.	6096.61	{5703., 6413.}	24.7982	0.12437	newly 3 weeks cumul	18981.	14607.5	{13670., 15285.}	23.0415	0.114301	newly 5 weeks spec	8590.	7620.8	{7269., 7918.}	11.2829	0.0523252	newly 5 weeks cumul	36334.	29304.4	{27513., 30582.}	19.3473	0.0938257	newly 56 weeks cumul	64192.	82760.6	{80948., 83807.}	28.9267	0.110281
40	newly 3 weeks spec	9375.	8494.28	{7455., 9737.}	12.2308	0.0582905	newly 3 weeks cumul	23924.	21717.4	{18486., 25573.}	14.693	0.0700099	newly 5 weeks spec	8935.	8555.49	{8073., 9010.}	4.67924	0.0210893	newly 5 weeks cumul	42535.	39138.9	{34745., 44226.}	11.0583	0.051932	newly 57 weeks cumul	74746.	79796.	{77661., 82204.}	6.75619	0.0282522
41	newly 3 weeks spec	9002.	8510.05	{7832., 9169.}	6.3818	0.0291536	newly 3 weeks cumul	21615.	20642.8	{19100., 22291.}	5.96687	0.027072	newly 5 weeks spec	8585.	9194.74	{8717., 9652.}	7.14432	0.0296614	newly 5 weeks cumul	39635.	39090.6	{36586., 41775.}	4.5345	0.0200008	newly 58 weeks cumul	64657.	82660.3	{75828., 86794.}	27.8443	0.105994
42	newly 3 weeks spec	13145.	7198.12	{6887., 7496.}	45.2406	0.261766	newly 3 weeks cumul	25907.	14882.6	{14333., 15520.}	42.5536	0.240918	newly 5 weeks spec	11817.	10455.5	{10182., 10784.}	11.5215	0.053338	newly 5 weeks cumul	52038.	34675.3	{33843., 35837.}	33.3654	0.176452	newly 62 weeks cumul	70332.	90922.8	{90956., 92155.}	29.2766	0.111337
43	newly 3 weeks spec	10255.	2594.58	{2507., 2678.}	74.6994	0.597024	newly 3 weeks cumul	22579.	6308.56	{6183., 6421.}	72.0601	0.553819	newly 5 weeks spec	11209.	3718.96	{3599., 3833.}	66.8217	0.479279	newly 5 weeks cumul	45260.	13193.1	{12959., 13426.}	70.8505	0.535418	newly 55 weeks cumul	87509.	73770.9	{73377., 74215.}	15.6991	0.0741724
44	newly 3 weeks spec	15502.	8827.89	{8639., 8985.}	43.0532	0.244591	newly 3 weeks cumul	33206.	19312.5	{18916., 19695.}	41.8404	0.235435	newly 5 weeks spec	12451.	10757.8	{10612., 10912.}	13.5991	0.0635076	newly 5 weeks cumul	61309.	40504.3	{39960., 41008.}	33.9342	0.180046	newly 61 weeks cumul	87960.	90910.9	{90710., 91098.}	3.35487	0.0143303
45	newly 3 weeks spec	16177.	6155.87	{5969., 6330.}	61.9468	0.419779	newly 3 weeks cumul	31598.	12802.1	{12460., 13096.}	59.4844	0.392502	newly 5 weeks spec	13010.	9524.01	{9360., 9710.}	26.7947	0.135527	newly 5 weeks cumul	61499.	30488.5	{29783., 31022.}	50.4243	0.304816	newly 60 weeks cumul	80329.	90829.5	{90546., 91116.}	13.0719	0.0533533
46	newly 3 weeks spec	10769.	5365.5	{4976., 5625.}	50.1764	0.303123	newly 3 weeks cumul	20202.	11132.7	{10337., 11642.}	44.8931	0.259369	newly 5 weeks spec	14108.	8776.32	{8295., 9078.}	37.7919	0.206397	newly 5 weeks cumul	48516.	27172.1	{25457., 28144.}	43.9936	0.252139	newly 60 weeks cumul	82843.	90396.8	{89790., 90903.}	9.11816	0.0378926
47	newly 3 weeks spec	12425.	7413.53	{7070., 7796.}	40.3338	0.224555	newly 3 weeks cumul	24055.	15375.5	{14604., 16178.}	36.0821	0.194738	newly 5 weeks spec	13477.	10544.4	{10382., 10757.}	21.76	0.106713	newly 5 weeks cumul	52391.	35409.7	{34087., 36710.}	32.4127	0.17031	newly 62 weeks cumul	79732.	90961.8	{90823., 92399.}	14.0845	0.0570331
48	newly 3 weeks spec	12536.	3403.87	{2253., 4856.}	72.8472	0.583983	newly 3 weeks cumul	21158.	6693.34	{4642., 9450.}	68.365	0.514656	newly 5 weeks spec	16548.	6929.3	{4999., 9403.}	58.1261	0.39259	newly 5 weeks cumul	54873.	18750.6	{13042., 26080.}	65.8291	0.481742	newly 62 weeks cumul	90499.	90118.1	{88350., 93009.}	2.33498	0.0102687
49	newly 3 weeks spec	21183.	4291.66	{2137., 7168.}	79.7401	0.739733	newly 3 weeks cumul	34718.	7781.58	{4259., 12420.}	77.5863	0.686296	newly 5 weeks spec	14957.	9061.95	{4929., 13667.}	39.4133	0.255575	newly 5 weeks cumul	71735.	23673.9	{12415., 37203.}	66.9981	0.521356	newly 64 weeks cumul	87984.	87097.1	{80461., 93651.}	6.92204	0.0306995
50	newly 3 weeks spec	10269.	5709.99	{5585., 5826.}	44.3959	0.254962	newly 3 weeks cumul	26490.	14236.3	{14055., 14390.}	46.258	0.269706	newly 5 weeks spec	9271.	6821.68	{6690., 6938.}	26.4192	0.133284	newly 5 weeks cumul	46170.	27494.9	{27201., 27778.}	40.4485	0.225123	newly 55 weeks cumul	81778.	78267.1	{77953., 78580.}	4.29321	0.0190594
